# Challenges in Healthcare Research in Nepal: What is the way forward?

**DOI:** 10.3126/nje.v15i2.75978

**Published:** 2025-12-31

**Authors:** Rajeeb Kumar Sah, Devendra Raj Singh, Bibha Simkhada, Vijay Singh GC, Padam Simkhada, Edwin van Teijlingen

**Affiliations:** 1 School of Human and Health Sciences, University of Huddersfield, Huddersfield, UK; 2 Centre for Health Economics, University of York, York, UK; 3 Centre for Midwifery & Women’s Health, Bournemouth University, Bournemouth, UK

## To The Editor:

Health research in Nepal has undergone significant changes over the last three decades, despite challenges, such as a lack of priority-driven research and skilled researchers, inadequate infrastructure, and unethical practices in research and publications. The Britain Nepal Academic Council (BNAC) 20^th^ Nepal Study Days (2023), hosted at the University of Huddersfield (UK), brought together stakeholders for a roundtable on examining key challenges in health research [1]. The discussions highlighted three key issues in Nepal: i) research ethics; ii) lack of validated and standardised research tools; and iii) researcher integrity and quality of publications.

## Research ethics

Over the years, the NHRC (Nepal Health Research Council) has played a central role in building research capacity, developing a research culture, and formalising the health research ethics review process and its implementation [2]. Despite these achievements, several challenges persist, including questions around the definition of health research, leaving uncertainty about whether research in areas such as sociology, health policy and systems, implementation studies, and socio-cultural and behavioural research falls under the existing NHRC ethical regulations, leaving researchers confused or unsure which studies require NHRC ethical approval [3]. Secondly, the current ethical review process appears to prioritise methodological aspects over core ethical principles such as autonomy, beneficence, justice, and/or nonmaleficence. Thirdly, reviews often focus on administrative procedures rather than rigorous ethical evaluation, which may be linked to a lack of suitable reviewers and/or unclear guidelines for selecting reviewers who are well-suited to the nature of the ethics application.

Thirdly, the NHRC continues to evaluate such ethics applications in implementation research through the lens of traditional public health/biomedical research, despite its need for adaptive methods [4]. Implementation studies often employ innovative, flexible, and practical research approaches based on the context and circumstances, where ethical principles cannot be applied mechanically, as in other general health research [5]. For example, the informed consent process may need to be modified for different stakeholders, including families, community members, leaders, and staff at government and non-governmental agencies [5,6]. Similarly, the risks and benefits of the study also need to be viewed within a specific context and time frame, rather than considering them from a clinical risk-benefit perspective [7]. To address these challenges, adequate awareness about the features of implementation research and its ethical implications is crucial for the ethics review team, both before the study, during its implementation, and in the post-study period.

## Lack of validated and standardised research tools

Selecting appropriate research tools is crucial to ensure the validity and reliability of the collected data. Researchers can use a validated and standardised questionnaire (instrument) or develop a new one; each has its strengths and weaknesses. Developing new tools requires extensive research, and it has to be validated and standardised before being used in data collection. This requires additional time and resources that can be constraints for many researchers and research projects, especially for low- and middle-income countries (LMICs) such as Nepal, where using validated and standardised questionnaires is are more practical option.

Validated and standardised tools ensure accuracy and consistency across research settings, populations, and times, enabling study comparisons and enhancing credibility and generalisability [8]. However, researchers must ensure the tools they use in their research are appropriate to the local sociocultural context. Nepal has made good progress in translating, adapting, and validating health-related tools such as the SF-36 and the WHO STEPS Survey, but many lack Nepali-language versions, prompting ad-hoc translations. Translation alone does not ensure validity; cross-cultural validation, which is a lengthy and complex process, including psychometric analysis, is essential to adapt to local nuances [9]. The NHRC can play an essential role in facilitating the cross-cultural validation and standardisation of the most commonly used research tools in the Nepali language. Such specific research technical issues must be complemented by promoting high quality research more generally.

## Researcher integrity and quality of publications

Research integrity and quality of publications ensure trust, credibility, validity, and reliability in scientific findings by promoting accountable researcher behaviour, which is crucial in resource-limited LMICs like Nepal to help foster responsible research practice. Academic organisations and research institutions must address barriers such as publication bias, plagiarism, conflicts of interest, data fabrication, inadequate peer review/research supervision, and overlapping publications to protect and preserve research integrity, which remain prevalent despite the University Grants Commission (UGC) and the NHRC efforts to curb misconduct [10].

UGC and NHRC play a pivotal role in promoting academic research by funding local educational institutions to conduct research and strengthen their research capacity, but the production of high quality research outputs for research-informed policy making is often lacking. Despite a seven-fold rise in Nepal’s health-related research publications (2008-2018) [11], there are rising concerns over quality, since many publications appear in low-quality or predatory journals, undermining research credibility [12]. Furthermore, only a small number of local health journals have an impact factor, possibly due to inexperience of journal editors and the editorial team. Though guidance exists, identifying predatory outlets can be challenging. UGC and NHRC should enforce guidelines, train researchers, and promote publishing in indexed databases, such as PubMed or SCOPUS.

## The Way Forward

NHRC could take the initiative to utilise the expertise of many Nepalese diasporic health researchers to act as ethical reviewers, journal editors, or seek their contribution through global research collaboration and capacity-building activities. We believe NHRC could formalise this by offering researchers unpaid positions as NHRC affiliates or honorary research associates, perhaps bringing in experienced researchers on its Advisory Board. Acknowledging researchers’ contributions through honorary positions and formal appreciation may motivate researchers to help promote and improve Nepal’s health research system over a longer period. Additionally, the NHRC could collaborate with and train Institutional Review Committees (IRCs) to understand their needs and build their capacity to streamline ethical oversight at local institutions.

Research integrity and publication quality could be enhanced by strengthening research ethics guidelines and regulations. Academic institutions should promote a research culture that values integrity and transparency and provide training and resources on research methodology and publication ethics. Establishing partnerships with international institutions will create platforms for knowledge sharing and research collaborations. Additionally, research institutions in Nepal should assist researchers in collecting data consistently and appropriately within the local context. We suggest that institutions like NHRC and UGC focus on resource development, including the identification, translation, or development of validated tools. These institutions could also play a leading role in facilitating the development of high-quality national journals and monitoring and improving the quality of local journals.

In today’s dynamic society it is important to make all aspects health research more relevant and its findings understandable for the general population and for politicians and policy-makers in particular. This means promoting all types of research methods, i.e. the ones most relevant to address the more complicated public health problems, but also being able to offer ethical review for the whole range of qualitative and quantitative research approaches. Nepal should clarify how these approaches can be appropriately integrated within the ethical regulations for health research.. As one of the key institutions responsible for research, the NHRC, in collaboration with local IRCs and other research-related institutions, should lead on the issues raised in this paper and improve both the quality of (a) research and (b) researchers in Nepal.

**Rajeeb Kumar Sah**, School of Human and Health Sciences, University of Huddersfield, Huddersfield, UK

**Devendra Raj Singh**, School of Human and Health Sciences, University of Huddersfield, Huddersfield, UK

**Bibha Simkhada**, School of Human and Health Sciences, University of Huddersfield, Huddersfield, UK

**Padam Simkhada**, School of Human and Health Sciences, University of Huddersfield, Huddersfield, UK

**Vijay Singh GC**, Centre for Health Economics, University of York, York, UK

**Edwin van Teijlingen**, Centre for Midwifery & Women’s Health, Bournemouth University, Bournemouth, UK

Dated: 10 March, 2025
